# Fagotti Score (FS) Compared with Peritoneal Cancer Index (PCI) for Preoperative Assessment of Optimal Cytoreduction in Upfront Laparoscopy

**DOI:** 10.3390/curroncol33080490

**Published:** 2026-08-20

**Authors:** Dimitrios Tsolakidis, Efthalia Markopoulou, Nikolaos Thomakos, Dimitris Haidopoulos, Dimitrios Zouzoulas, Vasilis Theodoulidis, Kimon Chatzistamatiou, Maria Topalidou, Eleni Timotheadou, Grigoris Grimbizis

**Affiliations:** 1 1st Department of Obstetrics and Gynecology, Aristotle University of Thessaloniki, “Papagergiou” Hospital, 56429 Thessaloniki, Greece; dtgyn@otenet.gr (D.T.); dzouzoulas@hotmail.gr (D.Z.); theodoulidisvasilis@yahoo.gr (V.T.); kimon.chatzistamatiou@gmail.com (K.C.); 2 1st Department of Obstetrics and Gynecology, School of Medicine, National and Kapodistrian University of Athens, Alexandra Hospital, 11528 Athens, Greece; thomakir@hotmail.com (N.T.); dimitrioshaidopoulos@gmail.com (D.H.); 3 Radiation Oncology, “Papageorgiou” Hospital, 56429 Thessaloniki, Greece; mariatop@gmail.com; 4 Department of Oncology, Aristotle University of Thessaloniki, “Papageorgiou” Hospital, 56429 Thessaloniki, Greece; timotheadou@auth.gr

**Keywords:** ovarian cancer, PCI, Fagotti score, resectability, laparoscopic PCI, PDS, IDS

## Abstract

**Background:** Ovarian cancer is the eighth most common cancer in women worldwide. Prognosis in ovarian cancer patients is mainly affected by the complete resectability of the tumor at the time of surgery. Among the methods used to determine resectability are laparotomy, laparoscopy, and imaging techniques. In ovarian cancer patients, the Fagotti Score (FS) and Peritoneal Cancer Index (PCI) can be used during upfront diagnostic laparoscopy to quantify disease burden, resectability, and prognosis. **Methods:** The aim of this retrospective study was to assess the FS and PCI as tools for predicting optimal cytoreduction (R = 0, <1) and correctly assigning patients for primary debulking surgery (PDS) or interval debulking surgery (IDS). An ROC was used to compare the accuracy of both FS and PCI scores for assessing resectability. Diagnostic measures for both were calculated. **Results:** A total of 45 patients were ultimately included in this study. The area under the curve was 0.801 for the FS and 0.705 for the laparoscopic PCI, with an optimal cut-off score of 24.5. In our cohort, the FS demonstrated a sensitivity of 90%, a specificity of 64%, a PPV of 66.7%, and an NPV of 88.9%. Similarly, using a cut-off of 24.5, the PCI demonstrated a sensitivity of 80%, a PPV of 59.3%, and an NPV of 56%. **Conclusions:** Laparoscopic PCI is a useful tool for predicting resectability in ovarian cancer patients. However, when compared with the PCI, it seems that the FS performs better as a predictive score for resectability.

## Simple Summary

Ovarian cancer is the eighth most common cancer in women worldwide. The prognosis for ovarian cancer patients is mainly affected by the stage of the disease at diagnosis and the ability to completely remove the tumor from the abdominal cavity during surgery. The ability to achieve complete removal of the tumor with no gross disease is called resectability. Resectability is assessed via imaging, diagnostic staging laparoscopy, and laparotomy using the ESMO-ESGO criteria, which evaluate nine parameters. Different scores exist to evaluate tumor resectability. During diagnostic staging laparoscopy, the Fagotti Score (FS) and Peritoneal Cancer Index (PCI) can be used to assess resectability. In our study, both the FS and PCI perform excellently as diagnostic tools for assessing resectability in ovarian cancer patients. Both Fagotti and PCI scores were highly accurate. The PCI score can be used as a prognostic marker for recurrence at 24 months; however, they cannot be used as prognostic factors for 5-year overall survival.

## 1. Introduction

Ovarian cancer is the eighth most common cancer and the eighth most common cause of cancer-related deaths in women worldwide. There are an estimated 20,890 new cases and 12,730 new deaths per year in the United States. Globally, in 2022, there were around 324,398 new cases and 206,839 deaths [1,2]. Notably, in women older than 45 years, 239,682 new cases were observed [3]. The incidence of ovarian cancer increased from 0.1 million to 0.2 million new cases per year, while total deaths reached 0.1 million; both figures increased by >80% between 1990 and 2021 [4,5].

Ovarian cancer is mainly of epithelial origin, with approximately 90% of all ovarian cancers being epithelial [1,6]. Non-epithelial ovarian cancers account for 10% of ovarian neoplasms and require different management and prognosis. In addition, the stage at diagnosis is a major determinant of survival (stage IV vs. stage I, RR = 10.54, 95% CI: 9.16–12.13), followed by histology, age at diagnosis, higher BMI, and smoking status [7]. Ovarian cancer tends to spread into the peritoneal cavity and is therefore diagnosed in an advanced stage in over >70% of cases [8]. Complete cytoreduction with no gross residual disease after surgery is an important prognostic factor [8].

According to current evidence, the optimal therapy for ovarian cancer is surgery and platinum-based chemotherapy. Debulking surgery typically includes total hysterectomy, salpingoophorectomy, pelvic and para-aortic enlarged lymph node dissection, omentectomy, and resection of all visible tumor load within the abdominal cavity [1,5]. Complete cytoreduction with no macroscopic disease (R = 0) is the most significant factor for progression-free survival (PFS) and overall survival (OS). If complete cytoreduction is not feasible during primary debulking surgery (PDS), optimal cytoreduction to macroscopic disease < 1 cm (R < 1) is preferred, and maximal effort to achieve residual disease of less than 1 cm is essential [9,10,11]. 

Primary debulking surgery (PDS) has been shown to provide a survival benefit in advanced ovarian cancer patients in large retrospective studies, despite the fact that three RCTs showed no survival benefit among patients who underwent primary debulking surgery or those who underwent interval debulking surgery after neoadjuvant chemotherapy [12]. A recent retrospective trial showed a significant improvement in survival of 15.2 months in patients who underwent PDS [9]. Recently, preliminary data from the TRUST trial, an RCT investigating the timing of debulking in advanced ovarian cancer patients, showed a benefit in PFS, but could not prove any benefit in OS [13]. In addition, a recent meta-analysis of all RCTs evaluating the timing of debulking showed no benefit of PDS over IDS in terms of PFS and OS [14]. A prerequisite for achieving this survival benefit is complete cytoreduction with no gross residual disease. Resectability in ovarian cancer patients is therefore the cornerstone of successful upfront debulking and an important determinant of survival [12,15]. Resectability is determined using the criteria described in the ESMO-ESGO consensus, based on the presence of nine markers, as described in Table 1 [10]. The main reason for unresectability is the major postoperative morbidity that follows radical resections in the intraabdominal areas, leading to adjuvant chemotherapy delays and subsequently to poorer survival rates. Recent studies have suggested that these criteria perform excellently in triaging patients, by imaging for primary debulking surgery (PDS) or neo-adjuvant chemotherapy [11,15].

Resectability is mainly assessed using imaging, laparoscopy, and laparotomy. However, there is no consensus on which is the best method. To date, there is no optimal imaging modality for assessing resectability. Laparotomy has been considered the gold standard in evaluating the extent of disease and tumor volume. However, diagnostic laparoscopy (DLS) before debulking surgery offers several advantages, such as assessing resectability through magnified visualization of the peritoneal cavity, shorter operating times, faster recovery, and earlier start of neoadjuvant chemotherapy [15,16]. This approach might spare patients from unnecessary staging laparotomies [16]. Furthermore, DLS appears to reduce futile laparotomies by 20% and is associated with a relative risk (RR) of 0.25 (95% CI: 0.13–0.47) for residual disease > 1 cm [15,16,17].

Several scoring systems exist to quantify the burden of disease and predict resectability. The Peritoneal Cancer Index quantifies disease extent in the abdominal cavity and can guide treatment options. It was first introduced by Sugarbaker et al. as a scoring system that incorporates tumor size and location within the abdominal cavity. Higher PCI scores are correlated with worse PFS and 5-year overall survival (OS) in advanced ovarian cancer patients [15]. 

In 2006, Fagotti et al. published a scoring system using a predictive index value (PIV) to predict the probability of optimal cytoreduction (R < 1) [18]. This scoring system evaluates the presence of disease in seven areas: omental cake, peritoneal carcinomatosis, diaphragmatic carcinomatosis, mesenteric retraction, bowel and stomach infiltration, and liver/spleen metastasis. These parameters provide an estimation of tumor load, and each parameter is assigned two points when present. A Fagotti Score ≥ 8 is usually a predictor of residual disease after upfront debulking in the majority of cases [15,16,19].

The primary aim of this study is to assess the PCI score as a tool for predicting resectability in patients with a pelvic mass or high suspicion of peritoneal malignancy and to compare it with the Fagotti Score. A secondary aim of this study is to determine whether these scores, assessed during upfront diagnostic laparoscopy, can predict progression-free survival (PFS) and overall survival (OS). An abstract of the preliminary data was presented at the 27th European Gynaecological Oncology Congress ESGO 2026 which was held in Copenhagen [20].

## 2. Materials and Methods

### 2.1. Study Characteristics

This is a single-center retrospective cohort study aimed at comparing the Fagotti Score with the PCI score as diagnostic tools for predicting resectability in ovarian cancer patients already assessed according to the ESGO-ESMO resectability criteria. All data were derived from the Papageorgiou General Hospital patient database, an ESGO-certified ovarian cancer center that performs 50–70 ovarian cancer operations per year. All files of patients who underwent Fagotti laparoscopy between 2017 and 2023 were retrieved and assessed, and all data were extracted from these files. 

### 2.2. Patient Characteristics

In this study, specific inclusion and exclusion criteria were set a priori. This study included only patients older than 18 years of age for whom there was high suspicion of primary or metastatic ovarian cancer or peritoneal malignancy. Patients were excluded from this study if they had a final diagnosis other than ovarian cancer, if they had other comorbidities that could undergo PDS by default, or if patients’ data were missing.

### 2.3. Clinical Data and Study Objectives

#### 2.3.1. Study Objectives

This study primarily aimed to evaluate the Peritoneal Cancer Index (PCI) as a predictive tool for resectability in patients presenting with a pelvic mass or a high suspicion of peritoneal malignancy, and to compare its performance with the Fagotti Score (FS). The secondary objective was to assess the prognostic value of both scores, as determined during diagnostic laparoscopy, in predicting survival outcomes in this patient population.

#### 2.3.2. Data Collection

Medical data were extracted manually through written and electronic records. Demographic, clinical, surgical, pathological, and follow-up data were collected, including the following: patient hospital identification number, patient age, ASA score, tumor biomarkers such as CA-125, FIGO stage, histological type, Fagotti score, PCI score, Aletti score, type of debulking (primary or interval), residual disease, type of debulking surgery performed for example PDS or IDS, neoadjuvant treatment, disease-free survival, and overall survival.

#### 2.3.3. Patient Initial Work-Up

During initial assessment, preoperative work-up included clinical examination, tumor marker CA-125 and all necessary imaging modalities for accurate initial diagnosis and staging. A CT scan of the thorax and abdomen and a pelvic MRI were requested for all patients. All cases were discussed in a multidisciplinary team meeting (MDT) and staged according to the ESGO-ESMO guidelines for ovarian cancer. For all cases, it was decided to perform diagnostic staging laparoscopy (DSL) (Fagotti laparoscopy) to assess tumor load extent, tumor resectability, and to obtain biopsies for final diagnosis.

#### 2.3.4. Surgery

All patients underwent DSL to assess resectability, and multiple tissue biopsies were obtained. In our center, the standard procedure is as follows: with the patient under general anesthesia in supine position, a 1 to 1.5 incision was made periumbilically (usually below the umbilicus). An open (Hasson) technique was used to establish pneumoperitoneum. The abdominal wall was opened in layers until the parietal peritoneum was exposed, which was then incised to enter the abdominal cavity through a 2 cm opening. A primary exploration of the abdominal cavity was performed using one finger to assess the mass, its adherence to the abdominal wall, and any other adhesions or obstructions. A central laparoscopic 10 mm trocar designed for this technique (Hasson trocar) was inserted, accompanied by a 0-degree laparoscopic scope to ensure safe entry. Then, the abdominal cavity was inflated with CO_2_ gas to induce pneumoperitoneum, after which one or two 5 mm peripheral trocars were inserted into the lower abdomen. If the typical positioning of the peripheral trocars is not possible, atypical positioning is acceptable to avoid rupture of the mass. A careful inspection of the abdominal cavity was performed to record the full tumor load, with all seven areas assessed for accurate calculation of the Fagotti Score (meticulous inspection of the small bowel serosa and mesentery, in order to rule out miliary disease). Representative biopsies were taken for histological confirmation and further molecular testing. At the end of the procedure, the abdominal cavity was deflated, all trocars were removed, and the umbilicus was closed in layers. All data and the final histological diagnosis were gathered, and the case was discussed again in an MDT meeting to determine the final diagnosis, tumor resectability, and further management. Primary debulking surgery was performed in patients who did not meet the ESGO-ESMO criteria for resectability or who had a PIV < 8.

Finally, residual disease was reported as R = 0 for complete debulking, R < 1 cm for optimal debulking, and R > 1 cm for suboptimal debulking in PDS patients. All interval debulking procedures were considered unresectable primarily by default.

#### 2.3.5. Scores

##### Fagotti Score

The Fagotti Score was calculated upon the evaluation of seven areas of the peritoneal cavity: omental cake requiring radical omentectomy, peritoneal parietal carcinomatosis, extensive diaphragmatic infiltration, mesenteric retraction, bowel infiltration, stomach infiltration and liver/spleen implants. These parameters received 2 points when present, and a total predictive index value (PIV) was calculated by two surgeons immediately after surgery [16]. 

##### Peritoneal Cancer Index Score

The Peritoneal Cancer Index was assessed via inspection of the peritoneal cavity during laparotomy or laparoscopy. The abdominal cavity was divided by two horizontal and two sagittal planes into 9 regions. Another 4 regions depict the small bowel. In total, 13 regions were evaluated for tumor load. All areas were given a score: 0 (no tumor seen), 1 (implants up to 0.5 cm), 2 (implants > 0.5 cm and less than 5 cm), or 3 (implants > 5 cm). The PCI score ranges from 0 to 39. Most PCI scores were calculated immediately after surgery. In cases where this step was omitted, the PCI score was calculated using the description in the operative report [21].

### 2.4. Statistical Analysis

Data are reported as continuous or discrete numerical variables and as ordinal categorical variables. For descriptive measures, data were assessed for their normality using the Kolmogorov–Smirnov test, and presented using mean, median, standard deviation, and interquartile range. The ROC was used to evaluate and compare the accuracy of both scores for predicting tumor resectability. An optimal cut-off value for the PCI score was calculated using the Youden Index. Sensitivity, specificity, positive predictive value (PPV), and negative predictive value (NPV) were calculated for both scores. For the Fagotti score, a cut-off value above 8 was used. Similarly, as no established cut-off for resectability exists for the PCI score, different cut-off values were used to calculate diagnostic accuracy measures. The most commonly used cut-off values of 15 and 20 were used for this analysis. The cut-off values that were extracted from the ROC curve analysis were also used. For time-to-event measures, 2-year progression-free (PFS) survival and 5-year overall survival (OS) were estimated from the day of surgery to the time of the event. The Kaplan–Meier method was used to perform survival analysis. 

All *p*-values reported are two-sided, and statistical significance was defined as *p* < 0.05.

All statistical analyses were performed with SPSS (version 29.0).

## 3. Results

### 3.1. Patient Characteristics

**Figure 1:**
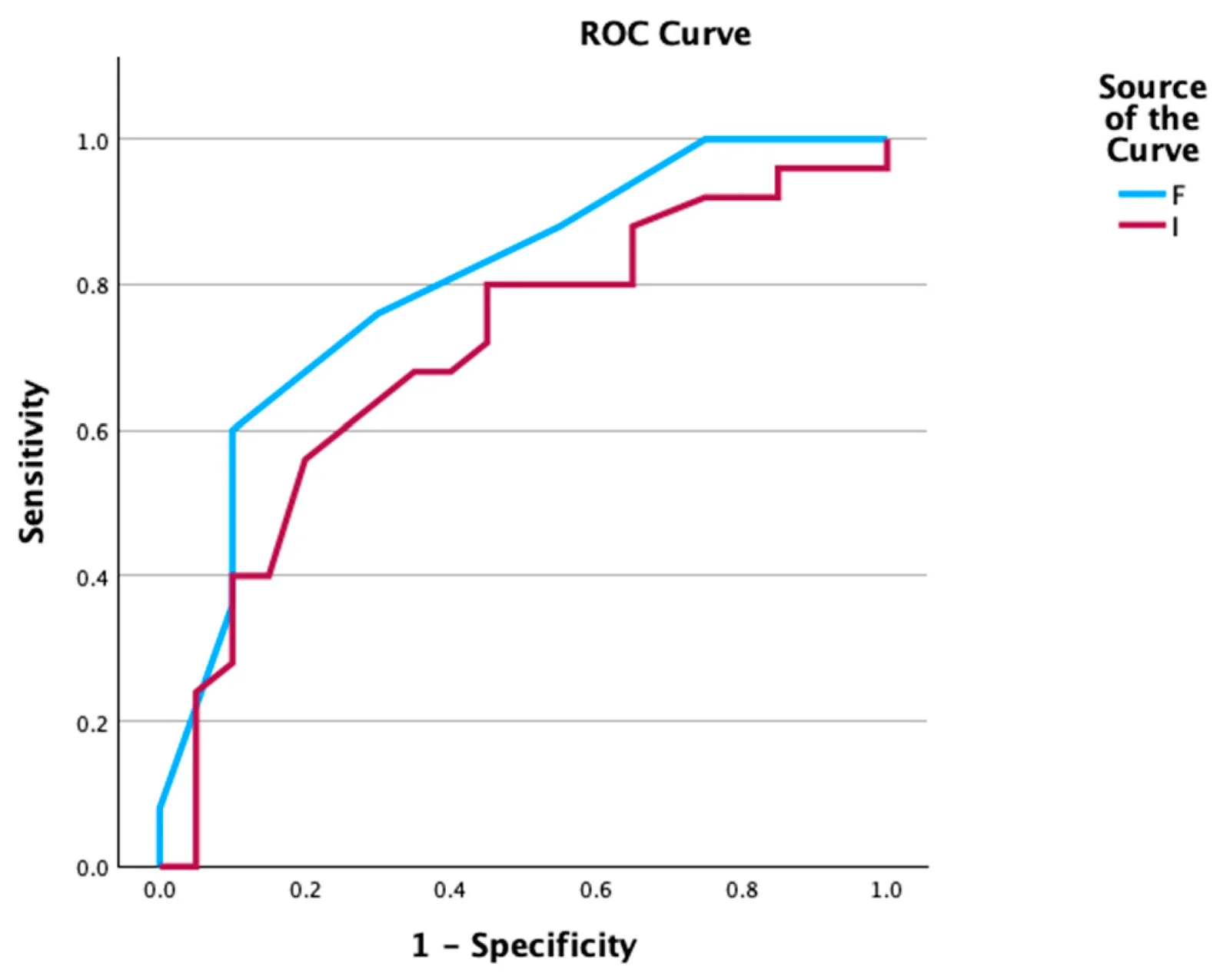
Receiver operating curve (ROC) comparing Fagotti Score (blue line) and PCI score (red line).

Between 2017 and 2023, 57 patients with peritoneal carcinomatosis underwent upfront laparoscopy in our center for resectability evaluation and histological confirmation of disease. From this cohort, four patients were excluded as the origin of the peritoneal malignancy was not ovarian (one endometrial cancer, one clear-cell endometrial cancer, one concomitant cervical cancer, one colorectal cancer), and eight patients were lost to follow-up or had missing data. Forty-five patients were finally included in the analysis (Figure 1). 

Patient characteristics are summarized in Table 2. 

The mean age of the patients was 61.58 ± 11.58 years old. Most patients had moderate comorbidities, with an American Society of Anesthesiologists (ASA) score of II (16/45 patients or 35.5%). Median Ca-125 in these patients was 749 (375.5–1750.7). The origin of peritoneal carcinomatosis was ovarian cancer in about 53/57 patients (93%). Most patients were staged as FIGO stage IV (21/45 or 46.6%), 18/45 (40%) as FIGO stage IIIC, 4/45 (9%) as FIGO stage IIIB, and 2/45 as FIGO stage II. Among these patients, 31/45 (69%) had serous histology, six (13.3%) had adenocarcinomas of unclear origin, four (8.9%) had endometrioid histology, and two (4.4%) had mucinous histology. 

The median Alleti Surgical Complexity score was 8 [IQR (5–9)], the median Fagotti Score was 6 [IQR (3–9)], and the median PCI score was 24 [IQR (16–27)]. 

In this cohort, 22/45 (49%) patients underwent PDS and 20/45 (44%) received NACT. Of those patients, three had disease progression or stable disease according to RECIST criteria and did not undergo IDS. Ultimately, 20/45 patients underwent IDS.

Complete or optimal cytoreduction (R = 0 or R < 1) was achieved in 17/22 (77%) patients who underwent PDS, and in 5/22 (23%) patients, suboptimal cytoreduction was achieved. All patients who underwent NACT were considered primarily unresectable. Details are shown in Table 2.

### 3.2. Primary Outcome: Diagnostic Accuracy Studies

Diagnostic accuracy measures were obtained, and the two scores were evaluated using ROCs. All measures are summarized in Table 3.

#### 3.2.1. Fagotti Score

All diagnostic measures were calculated for a Fagotti Score ≥ 8, with a sensitivity of 90%, a specificity of 64%, a PPV of 66.7%, and an NPV of 89.6%. The AUC for the FS was 0.801, with a 95% CI [0.670–0.932]. 

#### 3.2.2. PCI Score

For PCI scores, ROC analysis was used, and the AUC was calculated at 0.705, with 95% CI [0.549–0.861]. We used Youden’s index to determine the optimal cut-off value for our data. Between cut-off values of 23.5 and 24.5, we chose 24.5, as it provided better sensitivity, although lowering specificity. Four different cut-off values were used: 15, 20, 23.5, and 24.5. The corresponding sensitivity, specificity, PPVs, and NPVs are presented in Table 3. All ROCs are shown in Figure 1. It can be observed that the Fagotti Score performs better than the PCI score as a diagnostic tool for assessing resectability in our cohort.

### 3.3. Secondary Outcomes: Survival Analysis

A survival analysis was performed for our cohort using different cut-off values, and the prognostic significance of these values was established. Kaplan–Meier survival curves were created for both PFS with recurrence at 24 months and 5-year overall survival. 

Kaplan–Meier curves illustrated that a Fagotti Score ≥ 8 during DLS for patients with high suspicion of ovarian malignancy was not significantly correlated with PFS or OS. Moreover, different cut-off values for the PCI score were used in this survival analysis. 

**Figure 2:**
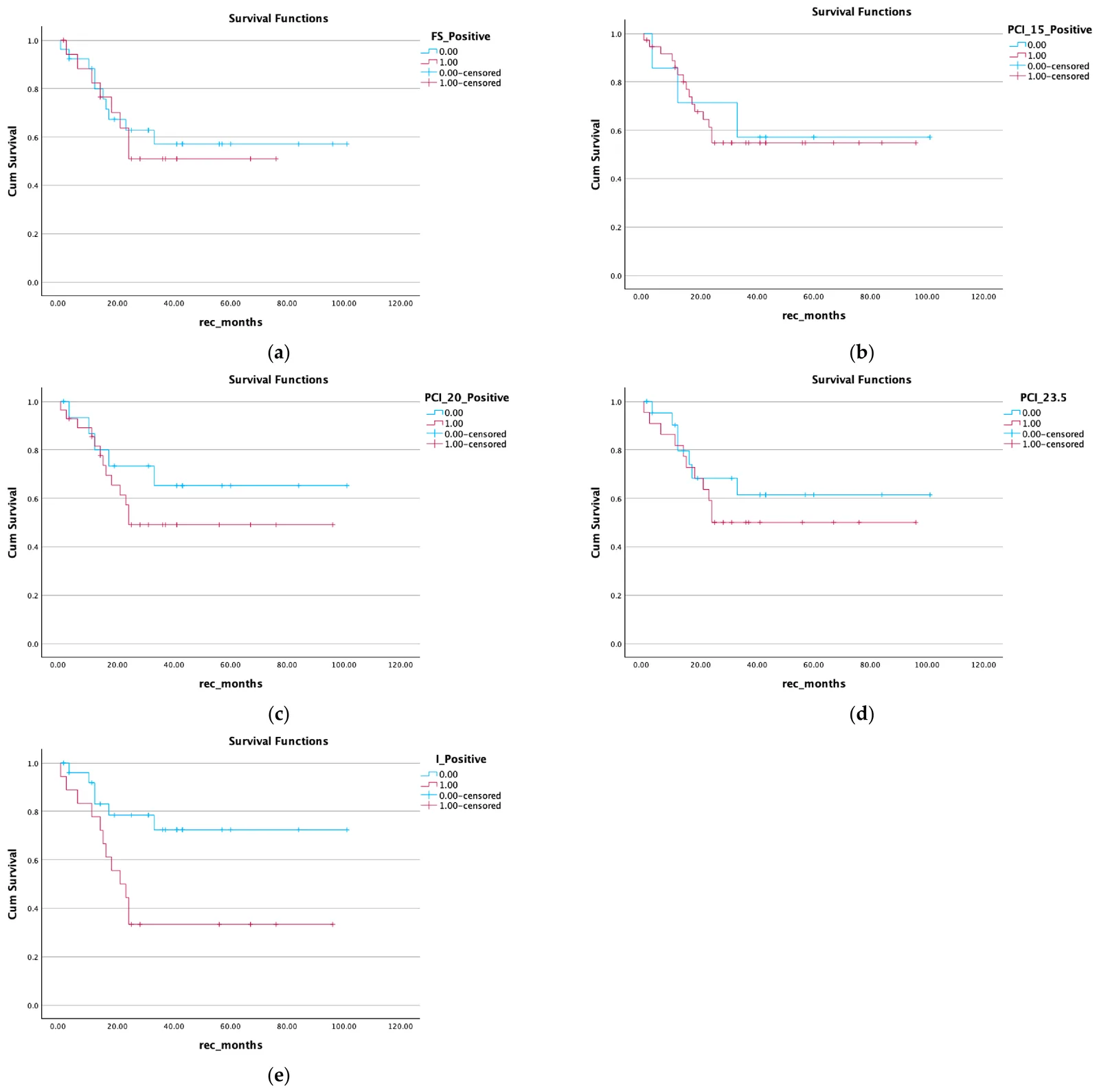
Progression-free survival for Fagotti Score ≥ 8 and for four different PCI values: (**a**) PFS for FS; (**b**) PFS for PCI ≥ 15; (**c**) PFS for PCI ≥ 20; (**d**) PFS for PCI ≥ 23.5; (**e**) PFS for PCI ≥ 24.5.

**Figure 3:**
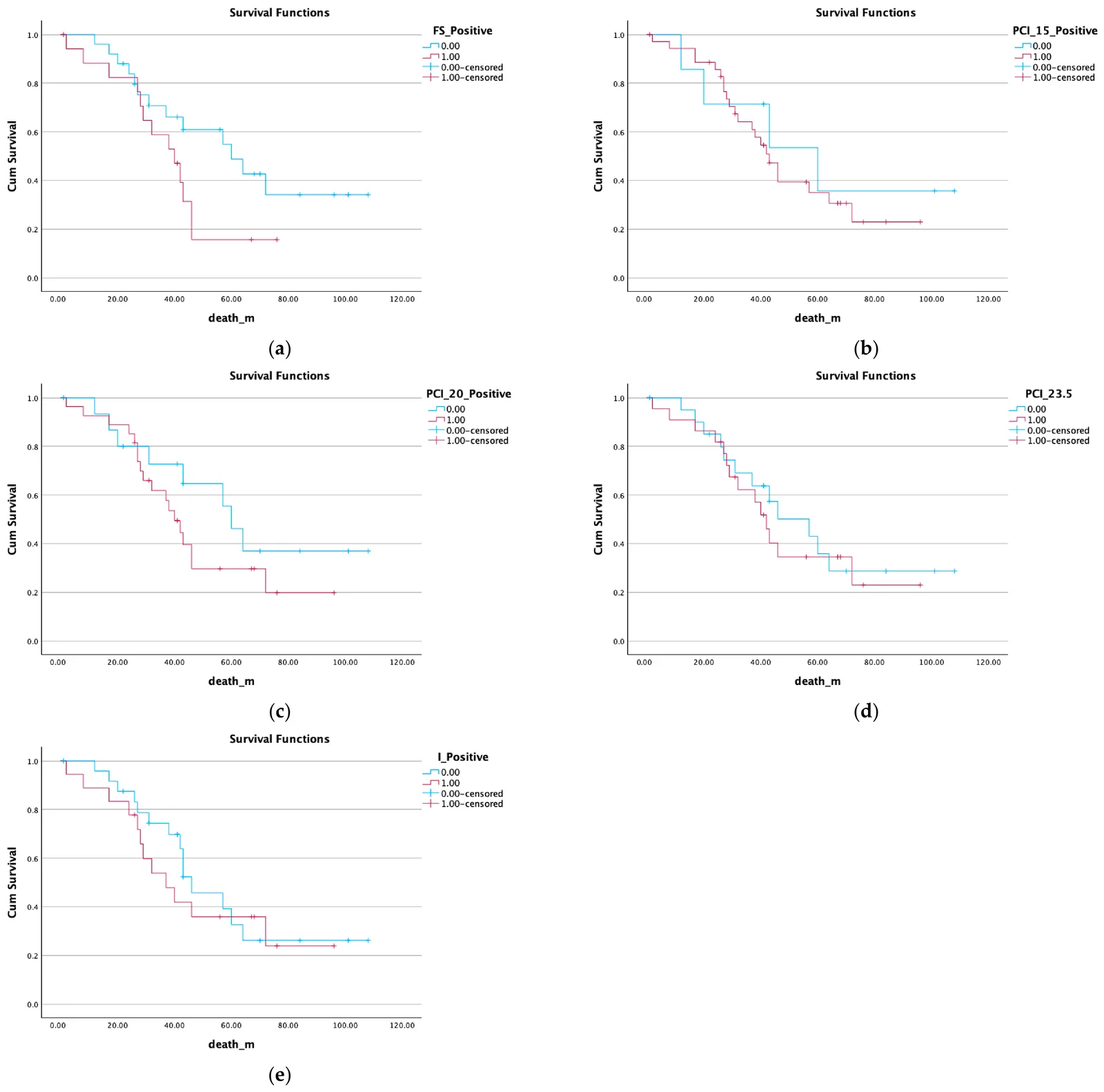
Overall survival for Fagotti Score ≥ 8 and for four different PCI values: (**a**) PFS for FS; (**b**) PFS for PCI ≥ 15; (**c**) PFS for PCI ≥ 20; (**d**) PFS for PCI ≥ 23.5; (**e**) PFS for PCI ≥ 24.5.

PCI cut-off values of 15, 20, and 23.5 were not correlated with disease-free survival at 24 months. Only a cut-off value of 24.5 was statistically significantly correlated with PFS at 24 months. For the same cut-off values, the laparoscopic PCI was not statistically significantly correlated with 5-year overall survival. These results are presented in Figure 2 and Figure 3. 

## 4. Discussion

Resectability is a major determinant of prognosis in ovarian cancer patients. Resectability can affect the course of treatment in ovarian cancer patients and may predict survival. Not assessing resectability correctly preoperatively can lead to suboptimal debulking and alter patient prognosis [15]. In fact, improved survival is achieved in patients with no gross residual disease after debulking surgery [22]. As no RCTs have shown a benefit in survival for patients undergoing PDS, and given that PDS may be associated with morbidity and mortality in advanced ovarian cancer patients, a balance should be kept when assigning patients to PDS [23]. Correctly assessing resectability before surgery may lead to better survival outcomes in ovarian cancer patients.

Among the methods used to predict resectability in these patients are preoperative imaging, diagnostic staging laparoscopy, and laparotomy [15,24,25]. Accordingly, different scoring systems have been developed, most of which have high sensitivities for predicting complete or optimal debulking surgery. These different systems either quantify disease-specific features that lead to suboptimal debulking or assess the extent and dissemination of disease [23]. 

Although laparotomy was considered the gold standard for evaluating resectability, laparoscopy should be incorporated as a triage tool for assigning patients correctly to either PDS or NACT. Laparoscopy may reduce unnecessary laparotomies resulting in suboptimal debulking, allow immediate initiation of neoadjuvant chemotherapy, and facilitate tissue sample collection for more accurate diagnosis [26]. In this study, the primary objective was to compare the Fagotti Score and PCI score as diagnostic tools for correctly assessing resectability during DLS. We also investigated their prognostic value in relation to PFS and OS, as a secondary objective.

In a recent Cochrane meta-analysis, the Fagotti Score with a cut-off ≥ 8 performed excellently as a diagnostic tool for predicting non-resectability [27]. For the two studies included in the meta-analysis, the sensitivities were 0.71 (95% CI: 0.44–0.88) and 0.95 (95% CI: 0.84–0.99). The NPV for the FS at this cut-off value ranged from 4 to 71%, and the AUC ranged from 0.66 to 0.98 [12]. In our cohort, the AUC for a Fagotti Score ≥ 8 was 0.801. 

Similarly, a systematic review that analyzed 19 studies evaluating preoperative scoring systems for advanced-stage ovarian cancer found that the average AUC for predicting optimal cytoreduction was 0.80, ranging from 0.63 to 0.98 [23]. Similarly, the performance of the PCI score for predicting non-resectability was also very good, with area under the receiver operating curve (AUC) values ranging from 0.69 to 0.94 [15]. 

In a retrospective study comparing the diagnostic accuracy of the laparoscopic PCI score to the Fagotti Score, the laparoscopic PCI score was reported as a more accurate measure for assessing tumor burden in peritoneal carcinomatosis. It is noteworthy that in this study, 14.7% of patients who had a final diagnosis of endometrial cancer were not excluded. These patients usually exhibit different courses, tumor progression, and tumor behavior [19]. In contrast, in our study, all patients with a final diagnosis other than ovarian and peritoneal cancer were excluded. Furthermore, the Fagotti Score was shown to be a more accurate tool for assessing resectability, with a greater area under the curve. 

A perioperative PCI >24 has been shown to be a strong predictor for suboptimal cytoreduction in ovarian cancer patients. In this study, a cut-off value of 24 reduced the frequency of incomplete CRS and major complications [28].

A PCI score obtained during laparotomy is probably more accurate in predicting resectability. In a retrospective study, it was found that the laparoscopic PCI underestimates the laparotomic PCI by approximately 2 points, and for every point that the laparoscopic PCI is increased, the laparotomic PCI is increased by one point [29]. Similarly, in a study by Gouy et al., the concordance of the laparoscopic PCI compared to that assessed during laparotomy is comparable. The mean difference observed was −2 (95% CI: −2.8 to −1.2) for a senior surgeon and −2.2 (95% CI: −3.1 to 1.3) for a junior surgeon [30]. 

Both the FS and PCI scores have also been readjusted for calculation with imaging. In the ISAAC study, a recent multicenter prospective study that examined both PIV and PCI scores calculated with imaging compared with surgery, very good concordance between these scores was shown [31,32]. Meanwhile, a recent study that compared radiologic, laparoscopic, and laparotomic PCI scores found that the laparoscopic PCI was the only independent predictor of complete cytoreduction [24]. 

Several factors influence survival in ovarian cancer patients, including lymph node involvement, age, cancer stage, performance status, and residual disease. Lymph node involvement and optimal cytoreduction are the most important prognostic factors for survival. In a meta-analysis by Yang et al. in 2023, which included studies with cut-off values mostly between 10 and 20, median survival was longer with PCI scores below the respective cut-off (56.7 months [45.2–68.2]) and shorter with those above cut-offs (28.8 months [95% CI: 23.0–24.6). Similar results were obtained in a sub-group analysis, as median survival was 73.9 months (52.0–95.8) with PCI cut-offs < 10 and 27.2 months (21.8, 32.6) with a PCI score > 10. Furthermore, survival was 66.6 months (95 % CI: 46.8, 86.4) with cut-offs below 8–12, but 28.3 months (95% CI: 15.0, 41.7) with cut-offs above 8–12. In this meta-analysis, higher PCI scores were associated with worse survival outcomes in a univariate (HR = 2.14; [95% CI: 1.63–2.66]) and multivariate analysis (HR 1.10; [95% CI: 1.02–1.180]) [33]. A more recent meta-analysis showed a significant correlation between the PCI and PFS (HR = 1.89; [95% CI: 1.51–2.36]) and between the PCI and OS (HR = 2.79; [95% CI: 2.04–3.82]). In our study, a PCI score with a cut-off ≥ 24.5, assessed during DLS, was shown to be statistically significant for PFS at 24 months. All other cut-off values were not significantly associated with PFS and OS in ovarian cancer patients [34]. 

In our study, a Fagotti Score ≥ 8 was not associated with reduced PFS and OS. On the contrary, a PCI value of 24.5 was associated with recurrence at 24 months. However, it was not found to alter survival prognosis. 

The main limitations of this study were its retrospective nature and the relatively low number of participants, as only 45 patients underwent upfront diagnostic laparoscopy in our department from 2017 to 2023. Although the results were produced through a robust statistical analysis, they should be interpreted with caution and generalizability should be very careful. Future larger, prospective studies are needed to verify these results.

## 5. Conclusions

In conclusion, both a Fagotti Score with a cut-off value ≥ 8 and a PCI score with an optimal cut-off value of 24.5 are excellent tools for predicting complete cytoreduction during laparoscopy. The Fagotti Score was shown to be more accurate in predicting resectability in ovarian cancer patients. Neither the Fagotti Score nor the PCI score was statistically significantly correlated with OS. A PCI score with a cut-off value ≥ 24.5 was correlated with shorter PFS at 24 months; however, it was not correlated with shorter 5-year OS.

## Figures and Tables

**Table 1:** ESMO-ESGO criteria for non-resectability.

ESMO-ESGO Markers for Non-Resectability
Diffuse carcinomatosis of the small bowel involving large segments such that resection would result in short bowel syndrome (remaining bowel < 1.5 m) Diffuse deep infiltration of the root of the small bowel mesentery Diffuse involvement/deep infiltration of: - Stomach/duodenum - Head or middle part of pancreas Involvement of coeliac trunk, hepatic arteries, or left gastric artery Central or multisegmental parenchymal liver metastases Multiple parenchymal lung metastases (preferably histologically proven) Non-resectable lymph nodes Brain metastases

**Table 2:** Patient characteristics.

Patient Characteristics	Mean ± SD	Percentage
**Age (mean)**	61.58 ± 11.58	
**ASA**		
ASA I	14/45	
ASA II	16/45	
ASA III	15/45	
Ca 125	749.2 (375.5–1750.7)	
**Stage**		
I	0	0%
II	2	4.40%
IIIA	0	0%
IIIB	4	9%
IIIC	18	40%
IV	21	46.60%
**Histology**		
Serous	31	69%
Mucinous	2	4.40%
Endometrioid	4	8.90%
Clear cell	2	4.40%
Adenocarcinomas (unclear origin)	6	13.30%
**Aletti Score**		
Low (<3)	0	
Intermediate (4–7)	17	
High (≥8)	18	
**Fagotti Score**	6 (IQR 3–9)	
**PCI Score**	24 (IQR 16–27)	
**Type of Debulking**		
PDS	22/45	49%
IDS	20/45	44%
**Residual Disease**	**PDS**	**IDS**
0	17	12
<1	4	8
>1	1	0

**Table 3:** Diagnostic accuracy of different laparoscopic scores and cut-off values.

Score	Sensitivity	Specificity	PPV	NPV
Fagotti Score ≥ 8	90%	64%	66.7%	88.9%
PCI				
≥15	25%	60.5%	71.4%	60.5%
≥20	55%	69%	55%	69%
≥23.5	65%	69.6%	59.1%	69.6%
≥24.5	80%	56%	59.3%	56%

## Data Availability

The original contributions presented in this study are included in the article. Further inquiries can be directed to the corresponding author.
